# Prosthetic Rehabilitation of a Non-optimally Positioned Implant With a Resin-Retained Restoration: A Clinical Case Report

**DOI:** 10.7759/cureus.60167

**Published:** 2024-05-12

**Authors:** Vedant Pathak, Seema Sathe, Anjali Bhoyar, Mithilesh Dhamande, Tanvi Jaiswal

**Affiliations:** 1 Prosthodontics, Crown, Bridge and Implantology, Sharad Pawar Dental College and Hospital, Datta Meghe Institute of Higher Education and Research, Wardha, IND

**Keywords:** recementation, decementation, maryland bridge, resin retained bridges, implant support restoration, resin bonded restoration

## Abstract

Long-term implant rehabilitation is still a problem. After about three months of implantation, the patient should go through rehabilitation to allow for osseointegration. However, poor patient cooperation during lengthy treatments or patient neglect, especially during patient-intensive treatments, can lead to a range of issues that require distinct approaches to solve. A fixed prosthetic is currently the most sought-after course of treatment. Implant misplacement can be a concern, especially during the prosthetic stage. Following the appropriate protocols, one of which has been discussed in this report, makes it possible to achieve prosthetic outcomes for a number of difficult cases. In the case described in this report, resin-retained restoration was used as an alternative to an implant-supported prosthesis because of the non-optimal position of the implant at the time of the prosthetic phase, which was due to discontinuation of treatment on the part of the patient. The conservative preparation of abutment teeth and pontic covering the non-optimally placed implant gave the best outcome and satisfaction on the part of the patient.

## Introduction

Prosthodontists strive for improved speech, contour, comfort, function, and stomatognathic system health in their modern dentistry practices. Implant-supported restorations are the suggested course of care for patients who are either completely or partially edentulous [1]. A five-year follow-up success rate of 97% has been reported in the literature for a single missing tooth restoration [1]. Osseointegration and the loading process are the two key factors influencing implant success [1]. Implant insertion problems can occur even with careful planning for the treatment plan. Three geometric planes, the implant's depth, position, and facial-palatal angulation, showcase the non-optimal implant position during the restorative phase of treatment planning [1]. Proper alignment in terms of length, angle, and placement is critical to the optimal implant-supported prosthesis. Proper osseointegration is not guaranteed by incorrect positioning, which increases the risk of failure and shortens the prosthesis lifespan [1].

Less invasive surgery is required for fixed prostheses known as resin-bonded bridges (RBBs) or resin-maintained bridges, which are held in place by composite resin cement. Since these restorations were first described in the 1970s, a great deal of progress has been made. The Rochette Bridge served as the prototype for the reinforced concrete beam, utilizing resin cement tags to ensure retention via a metal retainer featuring a distinct perforation [2]. Although methods were developed to try to improve micromechanical retention by altering the metal retainer's surface, the longevity of this type of restoration remained limited [2]. The name "Maryland Bridge" was created using an electrochemical etching technique developed at the University of Maryland. More recently, bridge retention has improved with the introduction of resin cement, which forms a chemical bond with both the metal alloy and the tooth surface [2]. RBBs preserve more tooth structure than traditional bridge preparations, which is the main advantage from a clinician's perspective [2].

Using a RBB can result in a fixed tooth replacement that is nearly reversible and protects the abutment tooth. For younger patients in particular, this is very important because extensive tooth preparation can raise the risk of endodontic complications [3]. The lack of long-term prospective success data has led to controversy surrounding the role of RBBs as definitive restorations, despite their acknowledged benefit. Most of the data comes from large, poorly controlled studies that used a range of cements and preparation methods [3]. As a result, it is challenging to identify the exact factors influencing the outcome. Based on recent systematic reviews, it has been observed that the five-year survival rates of bridgework differ based on design; conventional bridges have a survival rate of about 90%, while resin-bonded prostheses have an 87.7% rate [3].

Despite having a lower success rate over a five-year follow-up (94.5%) compared to implant-retained single crowns, RBB work still has benefits like less invasiveness, shorter treatment times overall, and lower costs [3]. Hussey et al. found high failure rates when evaluating the effectiveness of RBBs in general practice based on the amount of reimbursement fees claimed, in contrast to these optimistic assessments [3]. In addition, a recent study on dentists' use of RBB designs in hospital and general practice settings discovered that a significant percentage of practitioners employed unfavorable methods [3]. It would stand to reason that better planning and training could lead to better results. This report aims to revisit the role of RBBs in fixed prosthodontics and offer practitioners recommendations regarding bridge design, case selection, and clinical procedures to achieve successful outcomes [3].

## Case presentation

**Figure 1:**
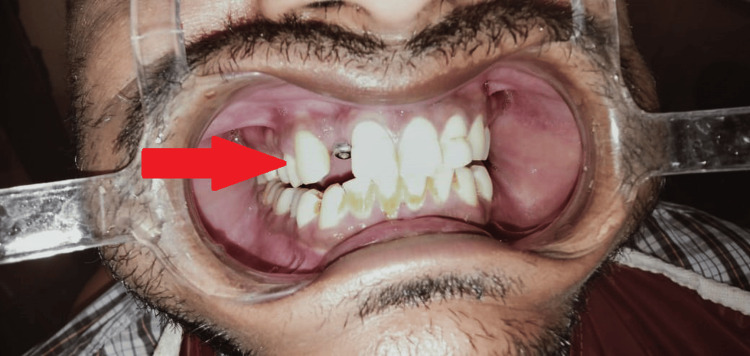
Pre-operative photograph showing malpositioned implant

A 36-year-old patient presented to the Department of Prosthodontics with the chief complaint of a missing maxillary right lateral incisor incompletely restored with an implant prosthesis placed 12 years back. The patient lacked motivation to complete the treatment after osseointegration (two to three months post implant placement) (Figure 1).

**Figure 2:**
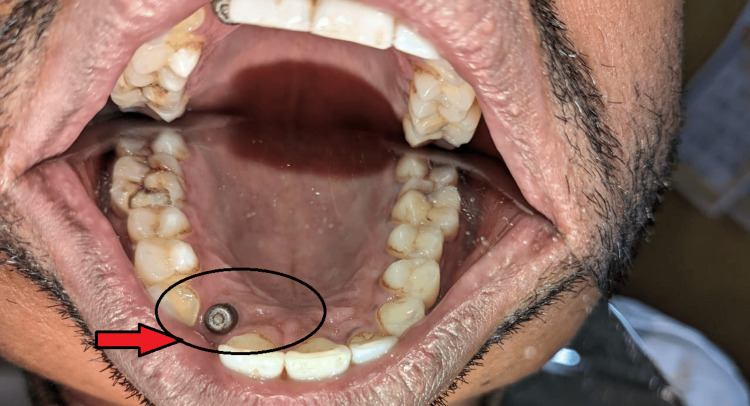
Relatively palatal migration of implant with time and discontinuation of the treatment

The patient also shows abfraction with maxillary, right and left central incisor, and left lateral incisor, which was treated by restoration in the Department of Conservative Dentistry and Endodontics. Due to the discontinuation of treatment in the last growth spurt by the patient, the implant had moved palatally (in comparison to the assumed initial position of the implant), also due to the long period the patient took for removal of gingival former from the implant screw, the latter had fused with each other (Figure 2).

**Figure 3:**
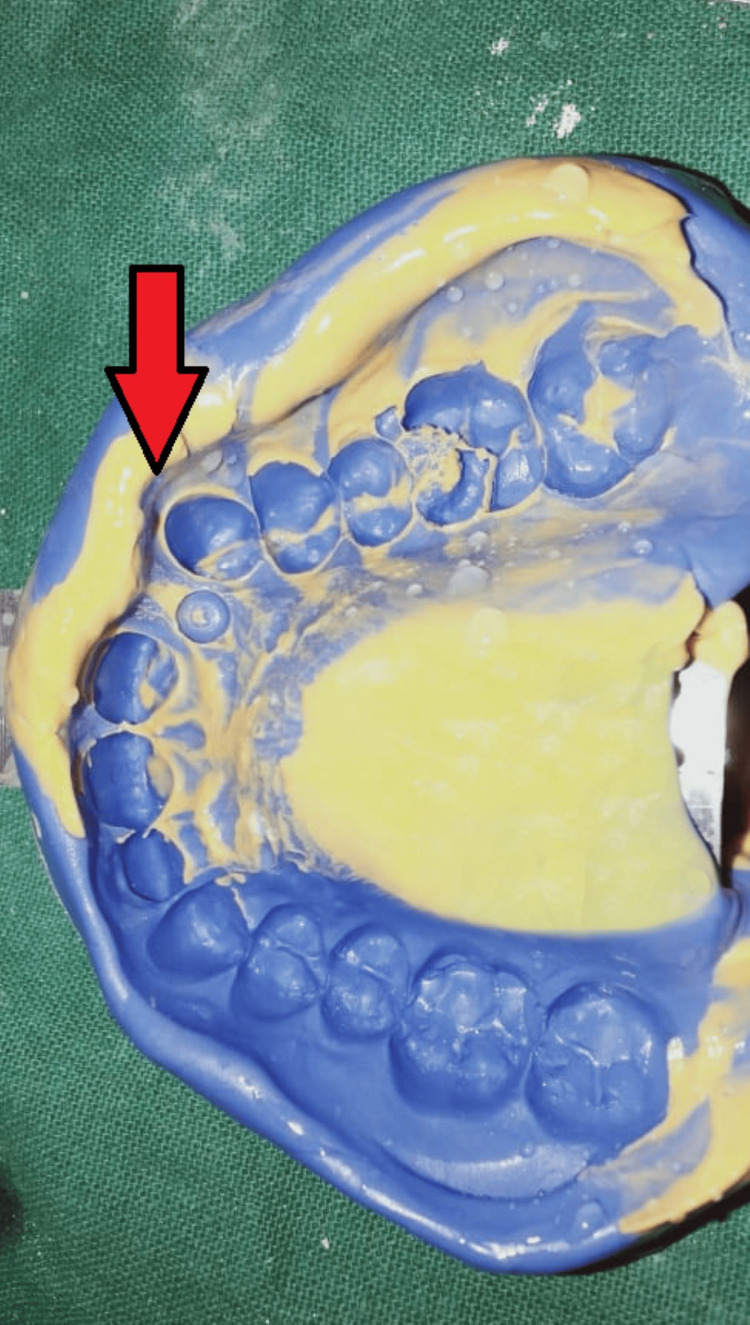
Elastomeric impression of prepared tooth

After consulting with the Department of Oral Surgery and by unwillingness of the patient to remove the malpositioned implant, it was decided to restore the missing maxillary right incisor by use of resin-retained restoration. Wing-shaped preparation was done on the maxillary right canine and right central incisor. Impression was recorded with elastomeric impression material (Figure 3).

**Figure 4:**
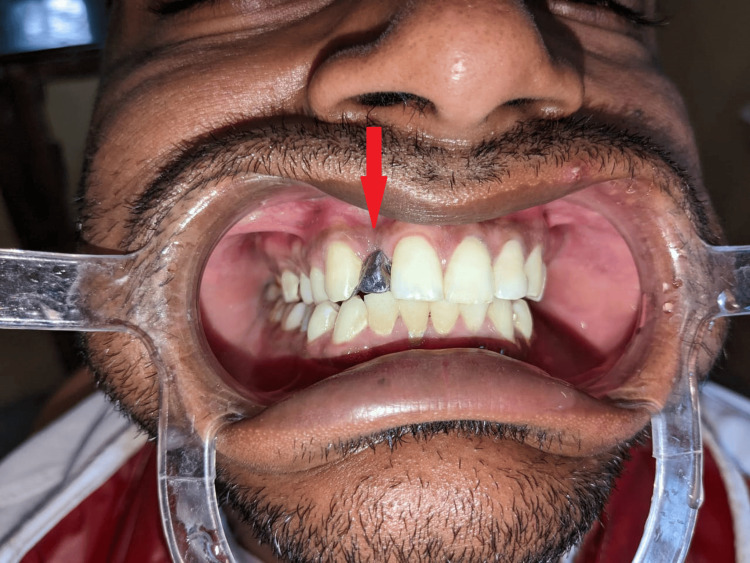
Metal try-in (frontal view)

**Figure 5:**
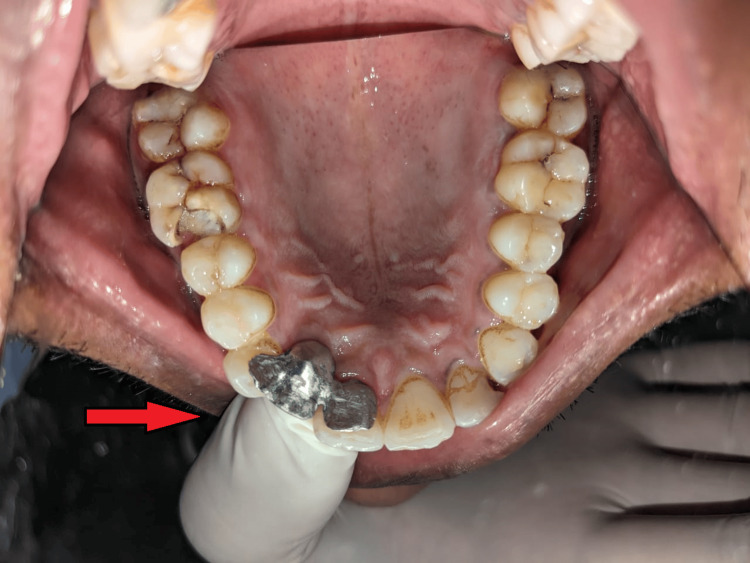
Metal try-in (occlusal view)

**Figure 6:**
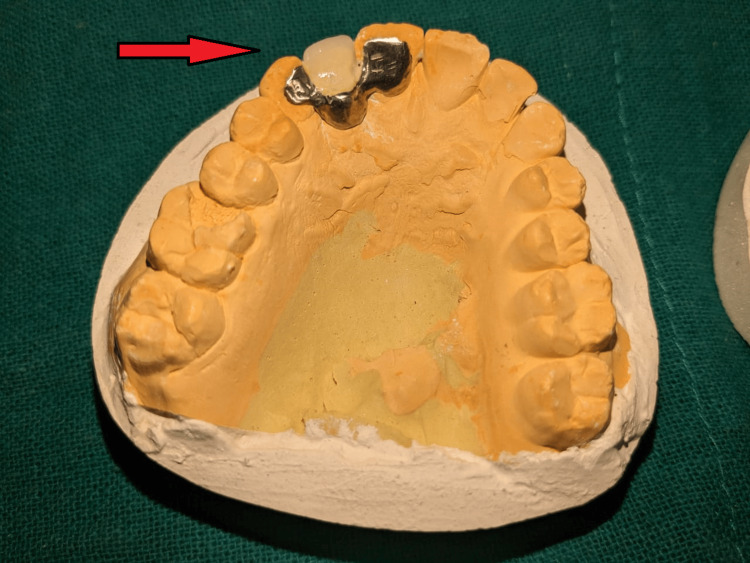
Fabrication of final prosthesis

**Figure 7:**
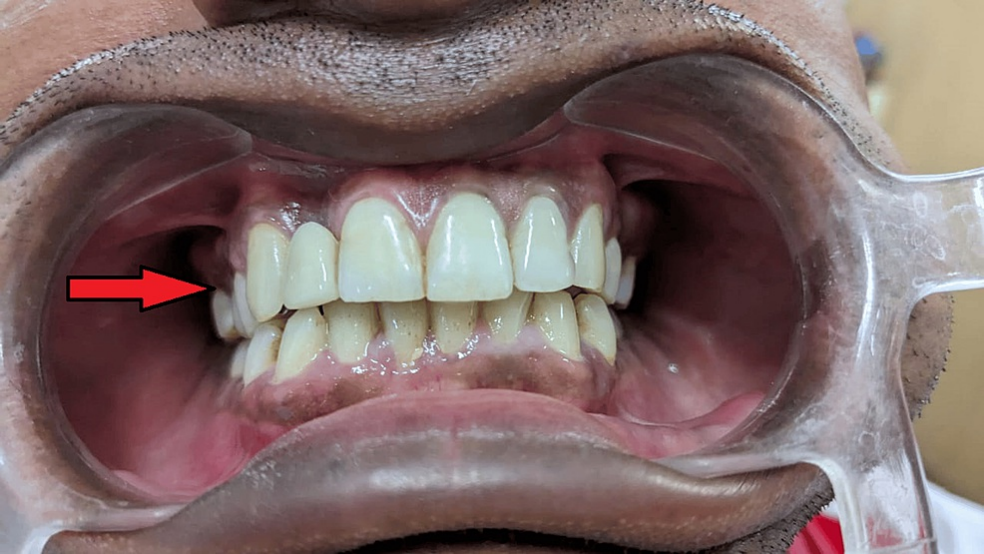
Cementation of final prosthesis

**Figure 8:**
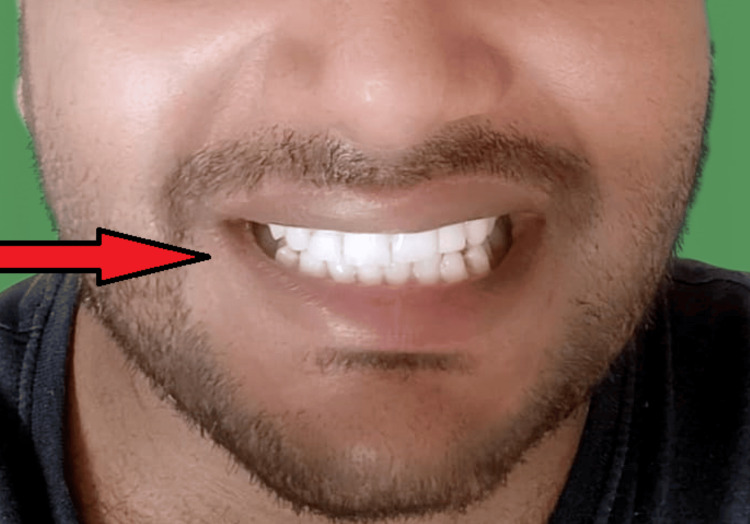
One-month follow-up picture (after treatment of abfraction of tooth with 11, 21, 22)

Metal try-in was done for approval from the patient for the positioning, shape, and size of the prosthesis (Figure 4, 5). The prosthesis was fabricated keeping in mind the shade and esthetics of the patient (Figure 6) and cemented with composite resin on the maxillary right canine and right central incisor (Figure 7). Though it is not a purely conventional resin-retained restoration, as it takes support from the implant too, this forms a novel approach as more coverage of oral structures gives more chances of retention of the prosthesis (by the implant). At the one-month follow-up to evaluate the esthetic acceptance of the patient (Figure 8), the patient was satisfied with the treatment. Later, caries in other teeth of the oral cavity were restored in the Department of Conservative Dentistry.

## Discussion

Although they don't happen very often, periodontal disease and dental caries are biological causes of Maryland bridge failure. [4] To avoid issues, oral health education should be completed after bridge cementation and should include guidance on diet, fluoride use, and oral hygiene. It should be given during treatment planning. Patients should be informed that there is a chance that one retainer may come loose and should report this right away if they think the bridge is loose. Debonding is the most frequent technical cause of Maryland bridge failure [5]. Remake or recement are the two options available when a bridge de-bonds. Regressing the restoration might be necessary if trauma caused the decementation. However, studies have shown that once a bridge has debonded, it is more likely to fail again [6], and recementing for a second time is generally ill-advised as replacing the bridge has been found to have a higher success rate [7]. This is most likely due to an innate issue with bridge design that occurs in most unsuccessful cases. With this in mind, the restoration itself should be examined and the patient should be reassessed from an occlusal perspective: Whether the patient has developed any parafunctional habit or any change in occlusion in inter-cuspal position or lateral excursion as a result of restoration or tooth wear of adjacent or opposing teeth should be investigated. Before attempting to re-cement a Maryland bridge, it is recommended to carefully remove any cement residue from the tooth and air abrade the metal retainer [8].

Because RBBs rarely require anesthesia and require little clinical time, they might be a good option for patients who are afraid of getting dental work done or cannot commit to more involved procedures requiring several appointments. Retention has been demonstrated to be impacted by the surface area that an RBB retainer covers. It is acknowledged that the ideal design for 180° wraparound retainers exists, but this needs to be balanced with the need for esthetics [9]. In order to increase surface area and enhance retention, retainers on posterior teeth may be extended to cover a portion of the occlusal surface as well as the palatal and lingual cusps. If required, crown lengthening can be done using electrosurgery or traditional periodontal flaps to maximize the surface area for bonding [10]. Since the patient in our case was not ready for additional surgical intervention, the long-standing osseointegrated implant was treated without removal. A conservative approach to replacing the lost tooth was required, and this was achieved by using a resin-retained restoration. After a month, the patient followed up, and while there was no evidence of a dislodgement issue, the patient was still advised to undergo oral prophylaxis and restorative treatment for carious teeth in the maxillary and mandibular arches.

## Conclusions

One of the main principles of tooth preparation for fixed prosthodontics is preserving tooth structure. This is the primary advantage of the RRB. Precision and attention to detail are just as important with RRBs as they are with conventional prosthetics. To provide a long-lasting prosthesis, the dentist must plan and fabricate a resin-retained restoration with the same level of care as conventional restorations. The method requires caution even though it can yield significant profits. One of the most important steps in predicting clinical success is careful patient selection.
